# Novel Activity of Oral Hypoglycemic Agents Linked with Decreased Formation of Tryptophan Metabolite, Kynurenic Acid

**DOI:** 10.3390/life14010127

**Published:** 2024-01-15

**Authors:** Kinga Bednarz, Kamila Kozieł, Ewa M. Urbańska

**Affiliations:** Laboratory of Cellular and Molecular Pharmacology, Chair and Department of Clinical and Experimental Pharmacology, Medical University, 20-090 Lublin, Poland; kbedna.98@gmail.com (K.B.);

**Keywords:** diabetes, metformin, glibenclamide, kynurenine pathway, inflammation, kynurenic acid

## Abstract

Kynurenic acid is a tryptophan (Trp) metabolite formed along the kynurenine (KYN) pathway in the brain and in peripheral tissues. The disturbed formation of kynurenic acid, which targets glutamate-mediated neurotransmission, GPR35, and aryl hydrocarbon receptors of immune or redox status, was implicated in the development of neuropsychiatric and metabolic disorders among others. Kynurenic acid exerts neuroprotective and immunomodulatory effects, yet its high brain levels may negatively impact cognition. Changes in the Trp–KYN pathway are also linked with the pathogenesis of diabetes mellitus, which is an established risk factor for cardiovascular and neurological diseases or cognitive deficits. Here, the effects of metformin and glibenclamide on the brain synthesis of kynurenic acid were evaluated. Acute exposure of rat cortical slices in vitro to either of the drugs reduced kynurenic acid production de novo. Glibenclamide, but not metformin, inhibited the activity of kynurenic acid biosynthetic enzymes, kynurenine aminotransferases (KATs) I and II, in semi-purified cortical homogenates. The reduced availability of kynurenic acid may be regarded as an unwanted effect, possibly alleviating the neuroprotective action of oral hypoglycemic agents. On the other hand, considering that both compounds ameliorate the cognitive deficits in animal and human studies and that high brain kynurenic acid may hamper learning and memory, its diminished synthesis may improve cognition.

## 1. Introduction

Tryptophan (Trp), an essential amino acid necessary for synthesis of peptides and proteins, is also a source of numerous biologically active compounds, such as indoles, serotonin, or kynurenines [1,2]. The Trp–kynurenine (KYN) pathway converts approximately 95% of Trp within the body [1,2]. Apart from the production of nicotinamide adenine dinucleotide, an essential cofactor of redox reactions, the pathway gives origin to various metabolites displaying a broad range of effects including immunomodulatory, antioxidant, neuroprotective or neurotoxic. Numerous research data support the potential role of disturbed Trp metabolism in the pathophysiology of several diseases including neuropsychiatric, cardiovascular, gastrointestinal or metabolic disorders [3,4].

Kynurenic acid is the product of Trp metabolism formed along the Trp–KYN pathway and showing neuroprotective as well as immunomodulating properties in various experimental models [3,4]. In the central nervous system, the formation of kynurenic acid occurs primarily in astrocytes from its bioprecursor, KYN, in the process of transamination carried out by KYN aminotransferases (KATs) I–IV. KAT II is considered the major enzyme responsible for kynurenic acid synthesis, producing approx. 75% of the compound at physiological conditions. Kynurenic acid was identified as an antagonist of excitatory amino acid receptors. It binds with the highest affinity with the glycine site of N-methyl-D-aspartate receptor (NMDA) and displaying lower affinity for the α-amino-3-hydroxy-5-methyl-4-isoxazolepropionic acid receptor (AMPA) and kainate receptor (KA) [5]. Kynurenic acid was also reported to act as an agonist of orphan receptors GPR35 and aryl hydrocarbon receptors, which have been recognized for their involvement in the modulation of immune response [6,7]. Additionally, kynurenic acid may act as a non-competitive inhibitor of α7 nicotinic cholinergic receptors, although available information is equivocal [8]. The antioxidant properties of kynurenic acid, which may act as a scavenger of free radicals, were also reported [9].

A number of in vitro and in vivo studies were performed in order to characterize the mechanisms regulating the synthesis of kynurenic acid. The activity of KATs and extracellular levels of kynurenic acid precursor, KYN, clearly determine the basal kynurenic acid formation in the brain and in the periphery [2,3,4]. Importantly, the activities of other Trp–KYN pathway enzymes, through the modulation of the KYN pool that can be used by KATs, are indirectly involved in the regulation of kynurenic acid levels [2,3,4]. A number of other factors that influence kynurenic acid formation were also identified. These include, e.g., the extracellular concentration of Na^+^, K^+^, and excitatory amino acids or the availability of oxygen and glucose [10]. Furthermore, pharmacotherapeutic agents, such as beta-adrenoceptor agonists, nitric oxide donors, memantine, antidepressants or some antiepileptics, were demonstrated to enhance the synthesis of kynurenic acid in the brain [11,12]. Conversely, non-steroidal anti-inflammatory agents and angiotensin converting enzyme inhibitors inhibit the synthesis of kynurenic acid [10].

Considerable evidence indicates that Trp derivatives produced along the Trp–KYN pathway influence various aspects of metabolism through their actions toward adipocytes, immune and muscle cells. The altered metabolism of the Trp–KYN pathway was implied as one of the pathogenic factors in the development of diabetes mellitus, which is a metabolic disease with increasing prevalence and high socioeconomic burden [13]. Long-term consequences of diabetes involve multiple organs dysfunction; moreover, diabetes mellitus is an established risk factor for cardiovascular and neurological diseases or cognitive deficits. Sub-acute or chronic inflammatory processes frequently detected in diabetic patients were demonstrated to impact the Trp–KYN pathway. On the contrary, Trp metabolites may modify the immune response, exerting either pro- or anti-inflammatory effects [14,15]. Under hyperglycemic conditions in vitro, the mitochondrial dysfunction and high concentrations of D,L-homocysteine inhibit the synthesis of brain kynurenic acid [16]. A study performed on rats with streptozocin-evoked diabetes mellitus revealed that the disease was linked with higher hippocampal, but not cortical or striatal, levels of kynurenic acid. Alterations in kynurenic acid production were accompanied by an enhanced KAT II activity in the cortex and hippocampus and normalized by insulin therapy yet only in cortical area [17]. Others reported an increased expression of indoleamine 2,3-dioxygenase (IDO), which is an enzyme degrading Trp and proposed as a regulator of immune response, in the hippocampus of diabetic rats [18].

Metformin is a synthetic biguanide administered orally in diabetes. The drug acts mostly through the inhibition of hepatic gluconeogenesis and is considered the first-line agent for a majority of diabetes mellitus type 2 patients [19]. Interestingly, metformin has been shown to exert beneficial effects beyond the modulation of glucose metabolism. Clinical interest in metformin ranges from its antitumor activity, through increasing life span by the alleviation of age-related inflammation, up to the therapy of neurodegenerative disorders [20]. Metformin activates the AMP-activated protein kinase (AMPK) pathway and inhibits the mTOR rapamycin kinase pathway with a subsequent enhancement of autophagy, reduced protein aggregation and improved mitochondrial function [20,21]. Metformin was also shown to limit reactive astrogliosis and to ameliorate the cognitive impairment [22]. A reduced risk of dementia among diabetic patients is supported by a number of clinical data [23]. Metformin increases the activity of neuronal Ser/Thr kinase, which, in the case of traumatic brain injury, may reduce the inflammatory reaction and improve cognitive functions after the injury [24].

Glibenclamide is a second-generation sulfonylurea derivative increasing insulin secretion by pancreatic β-cells. The compound interacts with sulfonylurea receptor (SUR) and demonstrates the ability to block various ion channels including K+-ATP channels in pancreatic and extra-pancreatic tissue [25]. Furthermore, glibenclamide blocks SUR 1 transient receptor potential melastatin 4 (SUR1-TRPM4) channels, which seems to underlie its ability to reduce brain edema [25,26]. Glibenclamide is considered an antidiabetic agent with promising neuroprotective properties that is able to maintain the integrity of the vascular endothelium, promoting neurogenesis and participating in anti-inflammatory processes [27].

We hypothesized that metformin and glibenclamide exert their neuroprotective effects partially through interference with kynurenic acid synthesis. Thus, the goal of this study was to investigate the potential effects of metformin and glibenclamide on the brain production of kynurenic acid under in vitro conditions.

## 2. Materials and Methods

### 2.1. Animals

The experiments were performed on the cortical brain tissue acquired from the brains of naïve adult Wistar rats, males, with body mass of 220 to 250 g, decapitated with all the required care and with maximally minimalized stress and pain. Animals were kept under standard laboratory conditions, at a 12 h light/dark cycle, 18 °C environmental temperature, with water and food available ad libitum. Obtaining the tissue from naïve animals which were not exposed to any procedures did not require special permission, according to the European Communities Council Directive on the use of animals in experimental studies and in agreement with the local guidelines (Local Ethics Committee for Animal Experiments in Lublin, Poland).

### 2.2. Materials

L-Kynurenine, pyridoxal-5′-phosphate, L-pyruvate, 2-mercaptoethanol, metformin, glibenclamide, cellulose membrane and ion exchange resin Dowex 50+ were purchased from Sigma-Aldrich (St. Louis, MO, USA). Water and other reagents used during high-performance liquid chromatography (HPLC) procedures were obtained from J.T. Baker Laboratory Chemicals.

### 2.3. Production of Kynurenic Acid In Vitro

The production of kynurenic acid de novo, under in vitro conditions in cortical slices, was studied as described in previous reports [28]. Briefly, rats were decapitated without prior anesthesia. The brains were quickly removed, and cerebral cortices were separated. Cortical slices, size 1 × 1 mm, were quickly prepared using McIlwain tissue chopper. Afterwards, slices were placed in a randomized manner within culture wells (Nunc 24-wells plates; 8 slices/well) prefilled with 900 µL of Krebs–Ringer buffer, pH 7.4, oxygenated for 30 min. Following a 10-min preincubation period, the solutions of tested substances were added in a volume of 50 µL. Subsequently, 50 µL of KYN solution, yielding a final concentration of 10 µM, was added to incubation wells. Blanks contained all ingredients of incubation media apart from brain slices. The incubation was carried out at 37 °C and lasted for 2 h. It was stopped by transferring culture wells into 1 °C water bath. The media were rapidly removed and mixed with 100 µL of 1N HCl and 20 µL of 50% trichloroacetic acid (TCA). Aggregated proteins were removed from the samples via centrifugation. The supernatant was subsequently applied on the analytical columns prefilled with cation-exchange resin (Dowex 50 W+) initially washed with 1 mL of Baker water and acidified using 1 mL of 0.1 N HCl. Next, 1 mL of HCl, followed by 1 mL of Baker water were applied to the columns. The fraction containing kynurenic acid was eluted using 3 mL of Baker water. Obtained samples containing kynurenic acid were stored at −72 °C until further analyses.

### 2.4. Analyses of the Activity of Kynurenine Aminotransferases I and II

The activity of KAT I and KAT II was assayed as described in earlier works, in semi-purified preparations [29]. Briefly, the cortical tissue was homogenized (1:9; wt:vol) in Tris-acetate buffer, which was composed of 10 mM 2-mercaptoethanol and 50 µM pyridoxal-phosphate, pH of 8.0. The final homogenate was dialyzed against 4L of the Tris-acetate buffer, for 16 h, at 8 °C. The enzymatic assays were carried out in the reaction mixture containing 100 µL of dialysate and 100 µL of the 150 mM Tris-acetate buffer at pH 9.5 for KAT I or 7.0 for KAT II, together with 2 µM L-KYN, 70 µM pyridoxal-5′phosphate and 1 mM pyruvate (final concentrations). The samples used for analyzing the activity of KAT II contained also 2 mM glutamine, which is an inhibitor of KAT I. Eight replicates were used for testing of each concentration, and every experiment was repeated three times. Blank samples contained the enzymatic dialysate that was inactivated with the use of high temperature (+95 °C). The incubation lasted for 2 h at 37 °C and was terminated by transferring samples to an ice bath. Then, 1 mL of 0.1 N HCl and 20 µL 50% TCA were added to samples, and clotted proteins were removed by centrifugation. Further procedures were performed as during the in vitro procedures.

### 2.5. Quantification of Kynurenic Acid

The content of kynurenic acid in samples from in vitro and enzymatic experiments was performed with the ultra-high-pressure liquid chromatography (UHPLC) method with fluorescence detection using Waters Acquity UHPLC system and Waters C18 analytical column, as previously described [30]. The mobile phase (20 mmol/L sodium acetate, 3 mmol/L zinc acetate and 7% acetonitrile) was run at a flow rate of 0.1 mL/min. The content of kynurenic acid in studied samples was carried out by a fluorescence detector with excitation at 344 nm and emission at 398 nm. The assay sensitivity reached 150 fmol of kynurenic acid/injection (signal:noise ratio = 5). During each assay, the calibration curve was calculated from external standards (0.2 pmol up to 1 pmol of kynurenic acid). The linearity of the calibration curve was not less than r^2^ > 0.999.

### 2.6. Statistical Analyses

The results were calculated as mean values and are presented on the graphs as a percentage of control values obtained for each experiment (considered 100%) ± standard deviation (SD). Statistica and GraphPad Prism v9.3.1. programs were used for statistical analyses. The statistical comparisons were performed by one-way analysis of variance (ANOVA) with a Bonferroni correction of *p* value. *p* value < 0.05 was considered statistically significant.

## 3. Results

### 3.1. Effect of Metformin and Glibenclamide on Kynurenic Acid Production In Vitro

**Figure 1:**
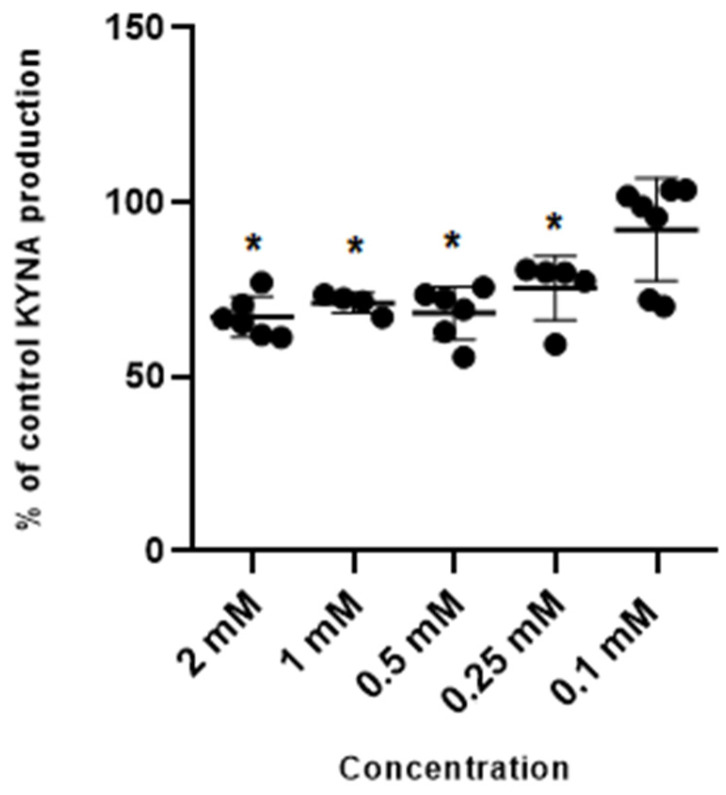
Effect of metformin on the cerebral production of kynurenic acid (KYNA) in vitro in cortical slices. Data are expressed as % of control values (100%) ± S.D. * *p* < 0.05 vs. control (ANOVA with Bonferroni adjustment of *p* value).

Metformin at concentrations of 2 mM, 1 mM, 0.5 mM and 0.25 mM inhibited kynurenic acid production in rat cortical slices in vitro to 66.65% ± 8.75; 71.15% ± 4.02; 67.99% ± 10.19 and 75.43% ± 12.04 of control, respectively (Figure 1). Metformin at 0.1 mM did not significantly affect the production of kynurenic acid.

**Figure 2:**
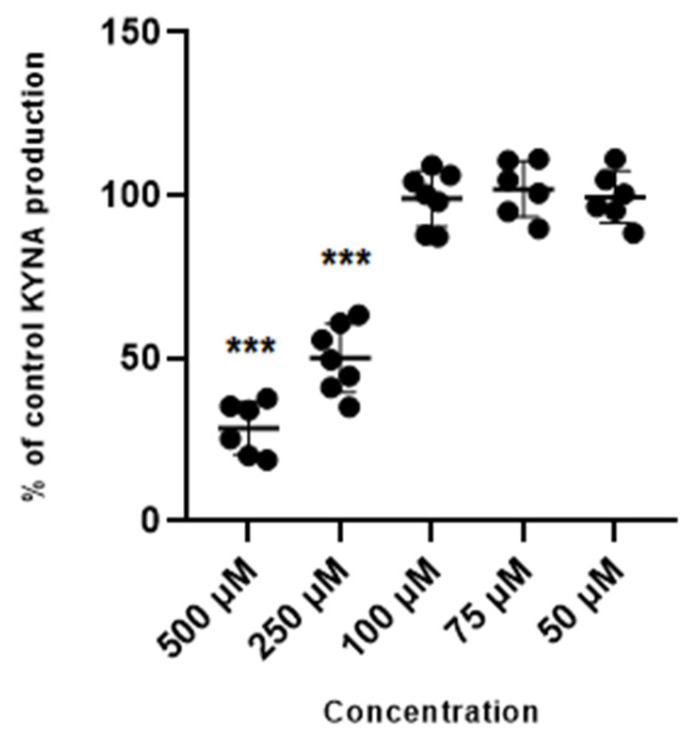
Effect of glibenclamide on the cerebral production of kynurenic acid (KYNA) in vitro in cortical slices. Data are expressed as % of control values (100%) ± S.D. *** *p* < 0.05 vs. control (ANOVA with Bonferroni adjustment of *p* value).

Glibenclamide used at 500 µM and 250 µM concentration reduced the production of kynurenic acid in vitro to 28.6% ± 8.2 and 50% ± 10.5 of control values. Glibenclamide at lower concentrations of 100 µM, 75 µM and 50 µM did not change the production of kynurenic acid in cortical slices in vitro (Figure 2). 

### 3.2. Effect of Metformin and Glibenclamide on the Activity of Kynurenine Aminotransferases (KATs) I and II

#### 3.2.1. Metformin

**Figure 3:**
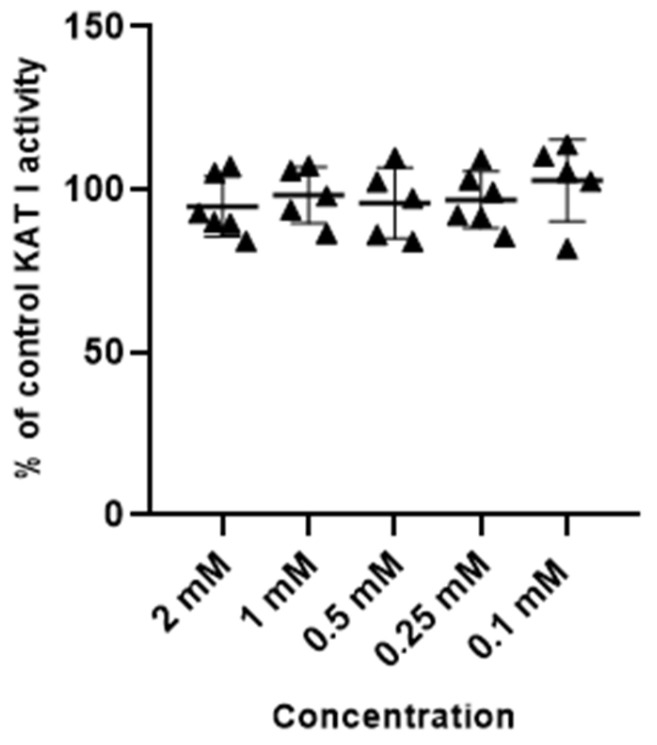
Effect of metformin on the activity of KAT I in semi-purified cortical tissue. Data are expressed as % of control values (100%) ± S.D.

**Figure 4:**
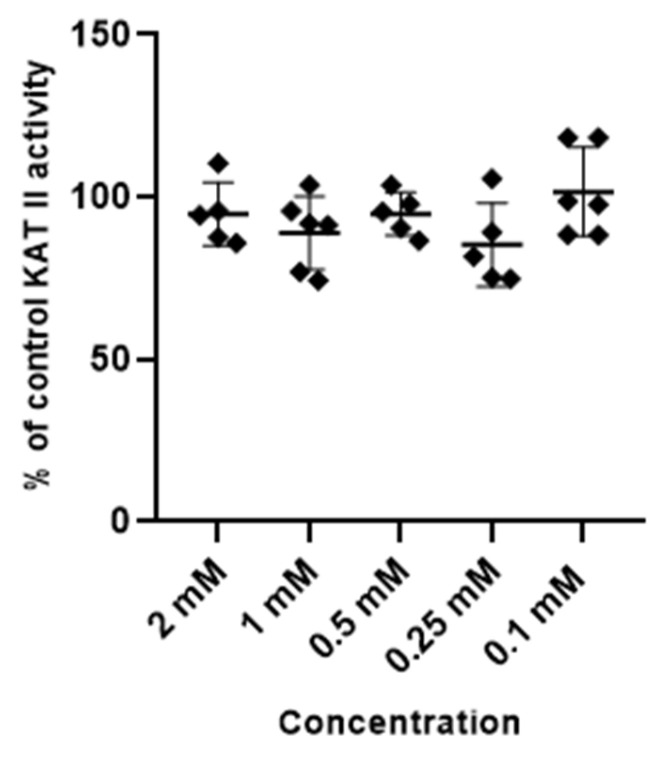
Effect of metformin on the activity of KAT II in semi-purified cortical tissue. Data are expressed as % of control values (100%) ± S.D.

Metformin at tested concentrations (2 mM; 1 mM; 0.5 mM; 0.25 mM and 0.1 mM) did not change the activity of enzymes synthesizing kynurenic acid, KAT I and KAT II, in semi-purified cortical homogenate (Figure 3 and Figure 4).

#### 3.2.2. Glibenclamide

**Figure 5:**
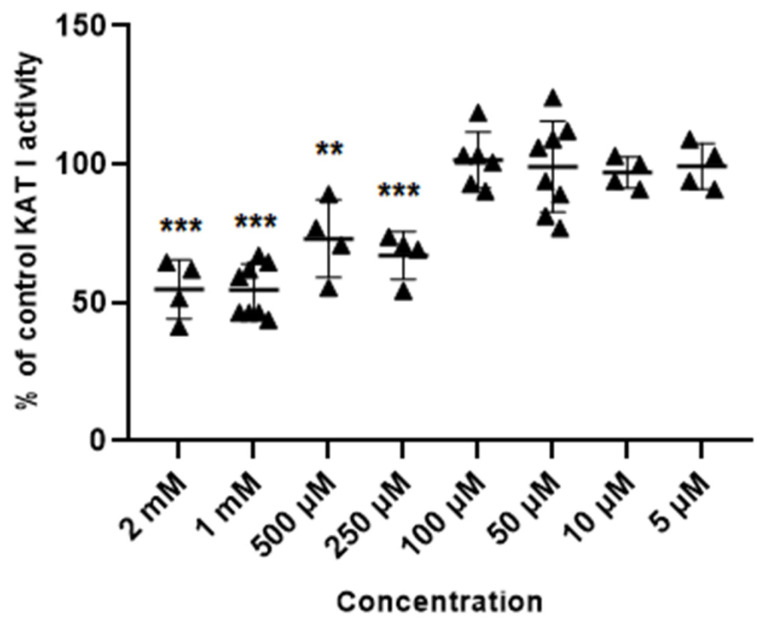
Effect of glibenclamide on the activity of KAT I in semi-purified cortical tissue. Data are expressed as % of control values (100%) ± S.D. ** *p* < 0.01; *** *p* < 0.001 vs. control (ANOVA with Bonferroni adjustment of *p* value).

Glibenclamide at concentrations of 2 mM, 1 mM, 0.5 mM and 0.25 mM inhibited the activity of KAT I to 54.8 ± 10.6; 54.5 ± 9.6; 73.1 ± 14.1 and 70.8% ± 6.2 of control, respectively. Glibenclamide at 100 µM, 50 µM, 10 µM and 5 µM did not affect the KAT I activity in semi-purified cortical homogenate (Figure 5).

**Figure 6:**
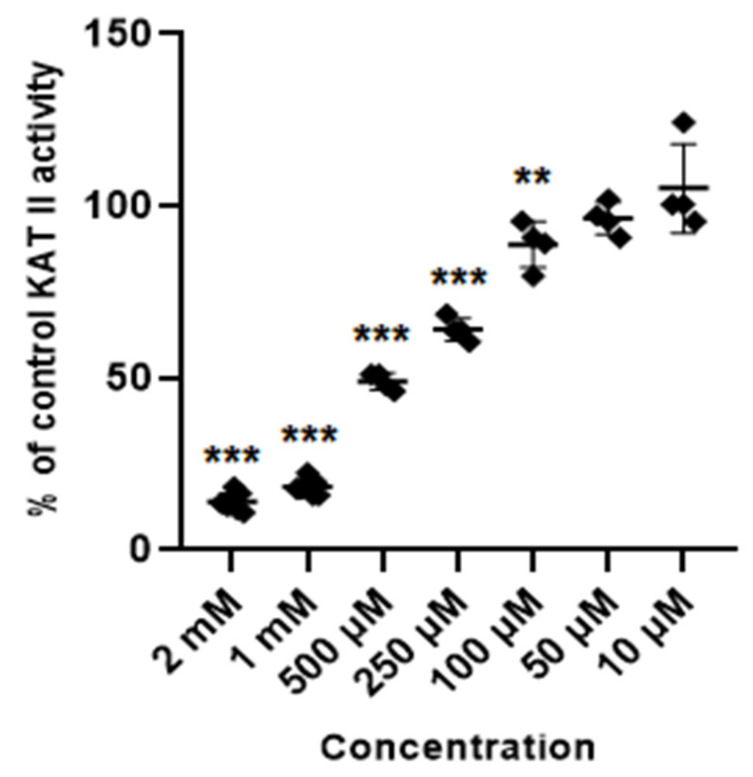
Effect of glibenclamide on the activity of KAT II in semi-purified cortical tissue. Data are mean values ± S.D. ** *p* < 0.01; *** *p* < 0.001 vs. control (ANOVA with Bonferroni adjustment of *p* value).

Glibenclamide at concentrations of 2 mM, 1 mM, 0.5 mM, 0.25 mM and 0.1 mM inhibited the activity of KAT II to 14% ± 2.4; 18.5% ± 2.1; 49% ± 2.4; 64.1% ± 3.3; and 88.8% ± 6.7 of control, respectively. Lower concentrations of glibenclamide (50 µM, 10 µM and 5 µM) did not significantly change the activity of KAT II in semi-purified cortical homogenate (Figure 6).

## 4. Discussion

The data presented here indicate that glibenclamide and metformin reduce kynurenic acid synthesis in rat cortical slices at concentrations of 250–500 µM and 250 µM–2 mM, respectively. The action of glibenclamide resulted most probably from the direct interference with the activity of KAT I and KAT II, which were inhibited by glibenclamide at concentrations of 250 µM–2 mM, and 100 µM–2 mM, respectively. In case of metformin, the observed reduction in kynurenic acid synthesis did not result from the changed activity of its biosynthetic enzymes.

Inhibitory effects of various agents on kynurenic acid synthesis were reported before. The angiotensin-converting enzyme inhibitor, ramipril, inhibits cortical kynurenic acid synthesis and the activity of KAT I and KAT II [31]. DL-homocysteine, a risk factor in atherosclerosis, acts biphasically and at low concentrations increases kynurenic acid production, while at high concentrations, it reduces kynurenic acid formation [32]. Angiotensin II receptor blockers, irbesartan, losartan and telmisartan, were shown to inhibit the production of kynurenic acid and to reduce the cortical activity of KAT II. Molecular docking suggests that their activity results from binding to the active site of KAT II [33]. Similarly, cyclooxygenase-2 inhibitors, niflumic acid and parecoxib block the active site of KAT II [34].

Accumulating data suggest that kynurenic acid may act as a molecule controlling energy use [35]. The underlying mechanism seems to depend on the activation of GPR35 receptors as well as on an enhanced phosphorylation of AMPK. This, in turn, may alleviate the inflammatory processes and reduce insulin resistance in adipocytes and muscle cells [35]. In contrast, other studies revealed that kynurenic acid and xanthurenic acid reduce the synthesis of proinsulin in isolated rat pancreatic islets [36]. Moreover, kynurenic acid may stimulate the secretion of glucose from hepatocytes indirectly in the mechanism linked with the block of NMDA receptors on the neurons of dorsal vagal nucleus [37]. On the other hand, it was demonstrated that plasma Trp and kynurenic acid levels are reduced early and before full manifestation of diabetes in animal models, i.e., in Spontaneously Diabetic Torii and Otsuka Long-Evans Tokushima Fatty rats [38]. In contrast, diabetic rhesus monkeys excrete 1.8-fold more kynurenic acid in comparison with healthy animals [39].

Previous studies in rat brain cortical slices demonstrated that the acute exposure of brain tissue to hyperglycemia does not affect kynurenic acid synthesis. Yet, hyperglycemia potentiates the reduction in kynurenic acid synthesis exerted by compounds inhibiting mitochondrial respiratory chain or by D,L-homocysteine. Thus, it was assumed that the mitochondrial dysfunction and hyperhomocysteinemia may act in concert with hyperglycemia, leading to central complications of diabetes [16]. Experiments conducted in vivo revealed that the kynurenic acid level is higher in the hippocampus but not in other studied brain regions of rats with experimental diabetes mellitus [17]. The altered hippocampal level of kynurenic acid was not modified by the therapy with insulin. No change in the activity of brain KAT I was observed. In contrast, the KAT II activity increased in the cortex and hippocampus of rats with diabetes. Insulin treatment normalized the cortical but not hippocampal KAT II activity [17].

Clinical data showed higher KYN and kynurenic acid levels in diabetic patients compared to the control [40]. Altered levels of Trp metabolites were also detected among diabetic patients with angina pectoris. Patients manifested higher levels of 3-hydroxykynurenine, KYN and kynurenic acid compared to patients with angina pectoris with normal glucose metabolism [41]. A small clinical study in two prediabetic subjects demonstrated that the metabolism of Trp toward kynurenines increases at the early stage of the disease [42]. Additionally, metformin was shown to reestablish the insulin sensitivity. This effect was paralleled by a decreased metabolism along the Trp–KYN pathway in responding subjects [42].

The data presented here corroborate the above findings, indicating that metformin may reduce central formation of kynurenic acid. Although the therapeutic concentration of metformin in the serum ranges between 5 and 20 µM, the level of metformin may reach from 250 to 680 µM among patients with lactic acidosis [43]. Thus, the changes observed here in kynurenic acid synthesis are clinically relevant yet seem to reflect mostly toxic and not therapeutic actions of metformin. However, during cerebrovascular events, the permeability of the blood–brain barrier increases, which enables the penetration of much higher quantities of metformin to the brain. Furthermore, we cannot exclude that in the brain, metformin is actively taken up by astrocytes and may reach high, micromolar concentrations. To our knowledge, this aspect of metformin action was not studied so far. The question arises regarding whether the observed effect is merely toxic or, potentially, could contribute to the cognitive improvement observed with metformin use. In fact, a decrease in brain kynurenic acid was demonstrated to alleviate memory deficits in experimental animals [44]. These results suggest that an impaired synthesis of kynurenic acid in the brain may be one of the factors contributing to the procognitive effects of metformin described in diabetic patients.

Further studies exploring the cellular mechanisms essential for the observed effects, including the analyses of potential contributions of the AMPK pathway, are needed. In fact, metformin treatment was shown to improve the cognition, reduce microglial and astrocytic activation, and to lower the synthesis of proinflammatory factors in aging mice [45]. Considering that glial cells are the most important source of kynurenic acid in the brain, the attenuation of astrocytic hypertrophy evoked by metformin can be one of the reasons for reduced kynurenic acid formation.

Glibenclamide, a sulfonylurea derivative, is used primarily as an oral antidiabetic agent in patients with DM type 2 and gestational diabetes due to its ability to stimulate insulin secretion in pancreatic β-cells [46,47]. Glibenclamide acts by binding to the SUR-1 subunit of the K+-ATP channel. Subsequently, closure of the potassium channel, depolarization of the membrane, influx of intracellular Ca^2+^, and a secretion of insulin follow [47].

Increasing evidence suggest that glibenclamide exerts pleiotropic actions in the central nervous system [25]. Glibenclamide utilized as an antidiabetic agent, when administered to patients with brain injuries, may ameliorate neuronal damage. One of the underlying mechanisms involves blocking of the SUR1-TRPM4 channels, which are up-regulated in neural and vascular cells as a result of ischemia or trauma, e.g., in the course of ischemic stroke, spinal cord injury or subarachnoid hemorrhage [48]. A glibenclamide-induced block of SUR1-TRPM4 channels seems beneficial in prevention of the post-stroke cerebral edema [26,49]. Furthermore, the administration of glibenclamide appears effective at reducing neuroinflammation and cognitive impairment associated with brain damage [50]. Postulated processes involved in this action include a decreased expression of proinflammatory molecules as well as improved neurogenesis and angiogenesis [51,52]. In rats, the chronic administration of glibenclamide ameliorated the development of spatial and working memory deficits and evoked an increase in hippocampal dopamine concentration accompanied by a decrease in dopamine and serotonin concentration in the striatum [53].

The therapeutic concentration of glibenclamide is low, reaching approx. 0.3–0.6 µM [54]. As it is with metformin, increased permeability of the blood–brain barrier after an ischemic insult improves glibenclamide penetration into the brain, yet the data on drug levels in the brain are ambiguous [26]. Glibenclamide evoked an inhibition of the synthesis of kynurenic acid and decreased the activity of KAT I and KAT II, which occurred at higher micromolar concentrations. When glibenclamide is used as a neuroprotective agent in brain injuries, lower kynurenic acid formation may potentially contribute to the reduction of beneficial effects of glibenclamide. Kynurenic acid, as an antagonist of glutamate NMDA receptors, shows neuroprotective activity in various experimental models. Glutamate excitotoxicity is a key mechanism leading to the brain damage after an ischemic stroke. The energy depletion, reduction in neuronal ATP synthesis, excessive glutamate release and decreased reuptake may result in an excessive activation of NMDA receptors, which in turn promotes neuronal cell death [55].

Indeed, some authors observed a lack of protective effect of glibenclamide. In the rat model of collagenase-induced intracerebral hemorrhage, glibenclamide neither reduced cerebral edema nor affected the permeability of the blood–brain barrier or restricted brain damage [56]. Similarly, in another rat model of acute intracerebral hemorrhage, no protective effect of glibenclamide was shown [57]. A limited efficacy of glibenclamide in cerebrovascular events has also been observed in some human studies [27].

## 5. Conclusions and Future Directions

The presented findings suggest that the reduction in brain synthesis of kynurenic acid may be involved in the activity of oral hypoglycemic agents, metformin and glibenclamide. In case of glibenclamide, but not of metformin, the effect seems to result from an inhibition of kynurenic acid biosynthetic enzymes, KAT I and KAT II. Bearing in mind that astrocytes are the major cells producing kynurenic acid in the brain, it can be hypothesized that a metformin-induced decrease in astrocytic hypertrophy can be one of the reasons for observed here reduction in kynurenic acid formation.

Although changes in kynurenic acid synthesis were observed at relatively high concentrations of studied drugs, it cannot be excluded that in case of a disrupted blood–brain barrier, both compounds may reach sufficient levels in the brain. In fact, loss of the blood–brain barrier integrity is a common pathology among DM patients and was implicated as an important factor in the development of dementia [58]. Furthermore, it is probable that during prolonged exposure to metformin and glibenclamide, the concentrations of drugs affecting brain kynurenic acid would be much lower.

A reduced availability of kynurenic acid may be regarded as an unwanted effect, possibly alleviating the neuroprotective action of oral hypoglycemic agents. On the other hand, considering that both compounds were shown to ameliorate the cognitive deficits in animal and human studies, and that excessive brain kynurenic acid may hamper learning and memory processes, its impaired synthesis may actually contribute to an improvement of cognition.

Further research under in vivo conditions and with longer exposure to the drugs is needed. Moreover, analyses of the potential action of other representatives of biguanides and sulfonylureas will help to clarify whether the observed effect is specific to the drugs studied here or represents the universal, novel mechanism of action of these two pharmacologic groups of drugs. It would be also of a great interest to evaluate the potential effects of metformin and glibenclamide on the function of the Trp–KYN pathway in peripheral tissues. The assessment of potential contributions of other mechanisms to the reduction in kynurenic acid synthesis, including the role of the AMPK pathway, performed under normo- and hyperglycemic conditions, should follow.

## Data Availability

The data presented in this study are available on request from the corresponding author.
